# Assessing evidence for avian-to-human transmission of influenza A/H9N2 virus in rural farming communities in northern Vietnam

**DOI:** 10.1099/jgv.0.000877

**Published:** 2017-08-04

**Authors:** Le Nguyen Minh Hoa, Nguyen Anh Tuan, Pham Ha My, Tran Thi Kieu Huong, Nguyen Thi Yen Chi, Trang Thi Hau Thu, Juan Carrique-Mas, Mai Thuy Duong, Nguyen Dang Tho, Nguyen Dang Hoang, To Long Thanh, Nguyen Thi Diep, Nguyen van Duong, Tran Khanh Toan, Trinh Son Tung, Le Quynh Mai, Munir Iqbal, Heiman Wertheim, H. Rogier van Doorn, Juliet E. Bryant, the VIZIONS consortium

**Affiliations:** ^1^​ Oxford University Clinical Research Unit, Hospital for Tropical Diseases, Vietnam; ^2^​ National Center for Veterinary Diagnostics, Hanoi, Vietnam; ^3^​ Department of Animal Health, Epidemiology Division, Hanoi, Vietnam; ^4^​ District Veterinary Services, BaVi District, Subdepartment of Animal Health, Hanoi province, Vietnam; ^5^​ Hanoi Medical University, Hanoi, Vietnam; ^6^​ National Institute Hygiene and Epidemiology, Hanoi, Vietnam; ^7^​ The Pirbright Insitute, UK; ^8^​ Radboud University, Nijmegen, Netherlands; ^9^​ Nuffield Department of Medicine, Centre for Tropical Medicine, University of Oxford, Oxford, UK

**Keywords:** avian influenza, H9N2, Vietnam, zoonoses, poultry, seroepidemiology

## Abstract

Rural farming communities in northern Vietnam do not routinely practice vaccination for influenza A viruses (IAV) for either humans or poultry, which enables us to study transmission intensity via seroepidemiology. Using samples from a longitudinal cohort of farming households, we determined the number of symptomatic and asymptomatic human infections for seasonal IAV and avian A/H9 over 2 years. As expected, we detected virologically confirmed acute cases of seasonal IAV in humans, as well as large numbers of subclinical seroconversions to A/H1pdm [55/265 (21 %)], A/H3 [95/265 (36 %)] and A/H9 [24/265 (9 %)]. Five of the A/H9 human seroconverters likely represented true infections rather than heterosubtypic immunity, because the individuals seroconverted solely to A/H9. Among co-located poultry, we found significantly higher seroprevalance for A/H5 compared to A/H9 in both chickens and ducks [for northern study sites overall, 337/1105 (30.5 %) seropositive for A/H5 and 123/1105 (11.1 %) seropositive for A/H9].

## Abbreviations

FAO, Food and Agriculture Organization of the United Nations; HPAI, highly pathogenic avian influenza; LBM, live bird market; LPAI, low pathogenicity avian influenza.

## Full-Text

Vietnam is considered to be a ‘hotspot’ for influenza A virus (IAV) evolution, both for human seasonal influenza and the emergence of animal IAVs with pandemic potential [1–3]. Highly pathogenic avian influenza (HPAI) A/H5 viruses have been endemic in Vietnamese poultry ever since the first major epizootic waves in 2005, which caused losses of >20 % of the standing poultry population at the time (45 million dead or destroyed) [4]. Mitigating the risk of pandemic influenza emergence has thus been a public health and animal health priority for over a decade, and substantial resources have been invested in all aspects of preparedness. A domestic manufacturing capacity for human influenza vaccines (for both seasonal and pandemic formulations) has recently been established, and licensure for the first made-in-Vietnam inactivated trivalent influenza vaccine (TIV) is anticipated for the end of 2017 [5]. It has been suggested that vaccination against seasonal IAVs among individuals at high risk for animal IAVs constitutes a fundamental tool for reducing the risk of co-infections, thereby reducing the potential evolution of novel IAVs by reassortment [6]. However, despite the high and sustained levels of A/H5 and A/H9 circulation in poultry [7, 8], as well as the high diversity of IAVs in swine (A/H1N1, A/H1N1pdm09 and A/H3N2 variants) [9], the last decade of intense surveillance has revealed a surprising dearth of animal-to-human IAV infections in Vietnam [10]. Given the impending availability of domestically produced human seasonal influenza vaccines in Vietnam, and the suggested high risk of emergence, there is significant motivation to better understand transmission ecology at the avian–human interface, and to systematically assess how individual immune profiles are shaped by past exposure and infection history.

Here we present a seroepidemiological study concerning low-pathogenic avian influenza (LPAI) A/H9 viruses among unvaccinated human and poultry populations in the backyard smallholder farms of Vietnam. We focused on LPAI A/H9 because a number of experimental investigations have confirmed direct-contact and airborne transmission of A/H9N2 among mammals, even in the absence of prior virus adaptation [11–14], while they have also shown that A/H9 contributed internal gene segments to lethal zoonotic infections with several subtypes, including A/H5N1, A/H7N9 and A/H10N8 [15–17], thus underscoring the credible pandemic threat posed by LPAI A/H9 viruses. On the global scale, there is accumulating evidence for widespread seroprevalence to LPAI A/H9 in both poultry-exposed and non-exposed 'general' populations [18], and thus there is continued uncertainty concerning whether the observed antibody titres for avian IAV reflect true infections or merely heterosubtypic immunity [19, 20]. Finally, in 2015 and 2016, despite the status of A/H9 as ‘low pathogenicity’, outbreaks of A/H9 associated with sudden death and substantial mortality in poultry were reported for the first time in northern Vietnam [8], suggesting possible changes to the local transmission ecology of avian IAVs that merited investigation.

We characterized IAV transmission within co-located human and poultry populations using both serological assays [classical haemagglutination inhibition (HI)] and virological screening of respiratory swabs. We determined human sero-reactivity to current seasonal IAVs (A/H1pdm and A/H3) and avian A/H9 among cohort members (*n*=265) sampled at three time points between 2013–2015. The cohort study design and sampling frame have been described elsewhere [21, 22]. Briefly, individuals were enrolled from farming households and livestock markets located in BaVi district, Hanoi province (a peri-urban area 60 km southwest of Hanoi city centre). After obtaining informed consent, serum and nasal/throat swabs were collected at enrollment (July to November 2013) to form a baseline, and at two annual cross-sectional resampling campaigns (in 2014 and 2015) (total samples=762). Participants were asked to report any acute febrile, respiratory or digestive signs, and project staff visited the homes to collect respiratory swabs within 48 h of symptom onset. None of the participants had a history of serious respiratory illness, and none had been vaccinated against seasonal influenza within the previous three years.

To better define concurrent IAV transmission within poultry sampled from the study sites, we also determined poultry seroprevalence to the predominant avian IAVs (A/H5 and A/H9) and screened oropharyngheal (OP) swabs collected from both apparently healthy and diseased poultry by RT-PCR. Poultry swab and serum collections were performed at the time of initial human enrolment, during annual cross-sectional resampling campaigns, and in response to human clinical episodes or reported animal disease. In addition to the co-located poultry samples from Hanoi, we had access to poultry sera from two distant provinces (DongThap and DakLak), and these were screened in parallel to the Hanoi sera. For study sites from Hanoi province (northern Vietnam), we were able to confirm that none of the farm flocks were vaccinated against A/H5 during the project period, although the vaccination status of poultry sampled from the two distant provinces was less clear.

HI assays were carried out using standard procedures [23], with twofold serial dilutions of sera mixed with 4 HA units of IAV antigen. The human IAV antigens comprised contemporary A/H1pdm and A/H3 antigens provided in the 2015 WHO reference kit. The avian A/H9 antigen was prepared from a recent Vietnamese H9N2 isolate (A/chicken/NgheAn_15AV13/2015) originating from a farm flock with large numbers of sudden death cases, representing the G1 lineage that is currently predominant in China and Vietnam [8]. Avian sera were screened against the same A/H9 antigen and A/H5 prepared from clade 2.3.2.1 .c (A/duck/Vietnam/NCVD-A2745/2013). The A/H5 antigen was inactivated with beta-propiolactone and biosafety-tested prior to use. HI assays on human sera were performed using standard pre-treatment with receptor-destroying enzyme (RDE) and turkey erythrocytes, whereas the HI assays on avian sera were tested directly, without RDE pre-treatment, and used chicken erythrocytes, as per standard OIE protocols employed at the National Centre for Veterinary Diagnostics [24]. We considered an HI antibody titre ≥1 : 40 as seropositive [25, 26], and defined seroconversion as a fourfold rise in titre. Virological screening of human respiratory swabs from clinical episodes used WHO/USCDC protocols for influenza A/B RT-PCR (http://www.who.int/influenza/resources/documents/molecular_diagnosis_influenza_virus_humans_update_201108.pdf). Virological screening of avian swabs was conducted on poultry swabs pooled by study site and species (maximum five swabs/pool), and processed as per national surveillance protocols [7].

**Fig. 1.**
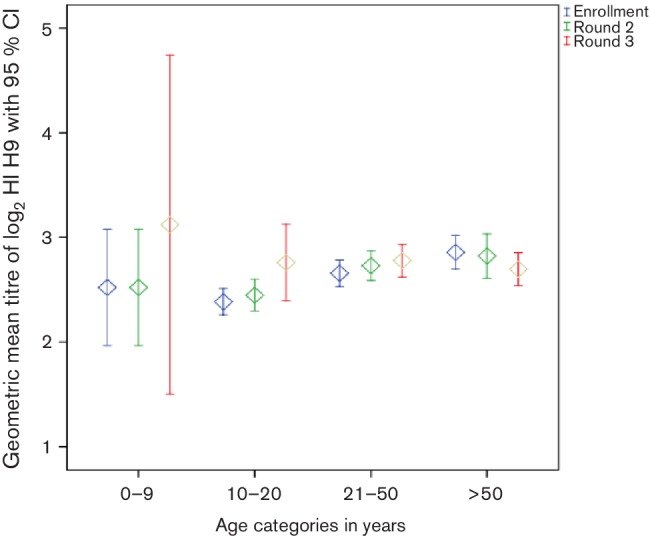
Geometric mean antibody titres (GMT) for A/H9 seropositivity at three time points, stratified by age group.

The demographic characteristics of the cohort members and a summary of the serological results are shown in Table 1. Using a cut-off threshold of 1 : 40 HI titre for seropositivity, an average of 3.5 % of participants had antibodies against A/H9N2 at each time point [5/256 (1.9 %) at enrolment; 9/263 (3.4 %) at year 1; 14/265 (5.3 %) at year 2] compared to the average seroprevelance of 58 and 39 % for A/H3 and A/HIpdm, respectively. Among the 265 participants, we detected 55 (21 %) seroconverters to A/H1pdm, 95 (36 %) seroconverters to A/H3 and 23 (8.7 %) seroconverters to A/H9N2 during the 2 years of follow-up. The median ages of the A/H1, A/H3 and A/H9 seroconverters were 40 (30–50), 38 (28.5–48) and 32 years (interquartile range, 21–39.5). Fig. 1 shows the A/H9 log_2_ geometric mean antibody titres (GMT) for the three serum collection time points (enrolment, year 1 and year 2) stratified by age group. Adults aged 21–50 years had the highest numbers of A/H9 seroconverters, while younger cohort members (<20 years old) showed greater trends towards elevated A/H9 titres in comparison to baseline, although the numbers within the lower age category were very limited. There was no clustering of H9 seroconversion by household. Among 23 H9 seroconverters, the majority demonstrated a titre fold change that was equivalent to or higher than that for seasonal IAV (5 individuals were triple seroconverters to A/H1pdm, A/H3 and A/H9; 4 were dual seroconverters to A/H1pdm and A/H9; and 9 were dual seroconverters to both A/H3 and A/H9). However, we observed five individuals who seroconverted to A/H9 alone. Interestingly, although none of the five individuals who were H9-only seroconverters reported clinical illnesses, two came from households that had experienced case clusters of respiratory illness, and one household also reported sudden death in poultry that was synchronous with the human illness (Table 2).

**Table 1.** Human seroprevalence and seroconversion data for the IAV antigens A/H1pdm, A/H3 and avian A/H9, stratified by age at enrolment. Seropositives were defined as HI titre ≥40 at each of the three sampling time points

				A/H1pdm positives (%)			A/H3 positives (%)			A/H9 positives (%)			**Seroconverters**		
**Age group (year)**	**No. of participants (%)**	**Median age, IQR (year)**	**Male sex (%)**	**Year 0**	**Year 1**	**Year 2**	**Year 0**	**Year 1**	**Year 2**	**Year 0**	**Year 1**	**Year 2**	**A/H1pdm**	**A/H3**	**A/H9**
0–9	6/265 (2.3)	8 (8)	2/6 (33)	2/5 (40)	2/6 (33)	4/6 (66)	3/5 (60)	6/6 (100)	5/6 (83)	0/5 (0)	0/6 (0)	1/6 (16)	1/6 (17)	2/6 (33)	1/6 (17)
10–20	36/265 (13.6)	15.5 (13–19)	14/36 (39)	19/33 (57)	25/36 (69)	18/35 (51)	26/33 (79)	30/36 (83)	30/35 (86)	0/33 (0)	0/36 (0)	4/35 (11)	8/36 (22)	14/36 (39)	4/36 (11)
21–50	158/265 (59.6)	38 (31–45.2)	62/158 (39)	63/154 (41)	72/157 (46)	70/145 (48)	80/154 (52)	99/157 (63)	90/145 (62)	5/154 (3)	7/157 (4)	9/145 (6)	33/158 (21)	61/158 (39)	16/158 (10)
>50	65/265 (24.5)	56 (53–70)	30/65 (46)	15/64 (23)	15/64 (23)	19/57 (33)	28/64 (44)	34/64 (53)	31/57 (54)	0/64 (0)	2/64 (3)	0/57 (0)	13/65 (20)	18/65 (28)	2/65 (3)
Overall	265	40 (27–50)	108 (40.8)	99/256 (38.6)	114/263 (43.3)	111/243 (45.6)	137/256 (53.5)	169/263 (64.2)	156/243 (64.2)	5/256 (1.9)	9/263 (3.4)	14/243 (5.7)	55/265 (20.7)	95/265 (35.8)	23/265 (8.6)

Previous studies of asymptomatic and mild avian IAV infections in humans observed that immune profiles typically yield low antibody titres that decrease quickly, and are often below the suggested threshold for clinical case definitions [27, 28]. We considered all individuals with a HI titre ≥40 to be positive, to be consistent with previous studies on A/H9 from the region [29, 30]. Using the HI ≥40 cutoff, our observed A/H9 seroprevalence among cohort members (3.5 %) was similar to that for poultry-exposed people in Tai’an, China [30], Shandong, China [29], and Cambodia [31]. The cut-off thresholds for seropositivity may critically influence interpretations, and both lower (HI ≥20) and higher thresholds (HI ≥80) have been used in the literature, complicating comparisons between studies and revealing the lack of consensus on appropriate interpretation of observed low antibody titres. One previous investigation of seasonal IAV among human populations in northern Vietnam also suggested that twofold changes in HI titre may actually correspond to true infections [32]. Our use of the fourfold criterion for seroconversion may thus underestimate the number of incident infections for both seasonal and avian IAV infections.

**Table 2.** Descriptive information for the five individuals who seroconverted to A/H9 but not seasonal antigens

**Cohort ID**	**Age**	**Gender**	**Enrolment study site**	**Testing of avians from the study site**	**Descriptive notes**
08–04	25	Female	Farming household	No avian serological or virological positives for A/H9, however all avian sera (*n*=5) from close-out were H5 seropositive.	Cohort member 08–04 did not report illness. However, clinical episodes were reported from other household members during the time interval of 08–04 A/H9 seroconversion. None of the sampled respiratory swabs from ILI-like cases in this household tested IAV positive by RT-PCR.
12–02	8	Female	Farming household	All avian sera (*n*=5) from each time point tested seropositive for both A/H5 and A/H9, but there were no virological positives.	This household had a brother–sister pair of A/H9 seroconverters. The brother (age 13 years) reported clinical symptoms, but nasal/throat swab was negative for IAV. He seroconverted to both A/H9 and A/H3. The sister (12-02) did not report symptoms and only seroconverted to A/H9.
58–03	73	Male	Farming household; family members slaughter livestock on the premises for retail sale	No avian seropositives or virological positives for A/H9, however all avian sera (*n*=6) from close-out were H5 seropositive.	Individual 58–03 did not report any illness episodes, however other family members reported sickness, and their clinical episodes were synchronous with illness in their livestock (cases of sudden death in avians). No virological confirmations of IAV from the sampled ILI cases. Other family members seroconverted either to seasonal antigens and/or A/H9, but none were H9-only seroconverters, except 58–03.
72–11	17	Female	Restaurant that maintained and slaughtered livestock on the premises, including both diverse avian species, local 'wild' pig breeds, and buffalo	All avian sera (*n*=5) from each time point tested positive for both A/H5 and A/H9.	Individual 72–11 did not report any illness episodes. She was not sampled at enrolment. Her year 1 and 2 sera were both seropositive for A/H3 (HI titres 640 and 320, respectively), indicating a previous infection with seasonal influenza in the year prior to sampling. She seroconverted to A/H9 with HI titre 5 to 40 between years 1 and 2. Neither of the two other restaurant workers recruited from that site seroconverted to any antigens.
73–03	22	Female	Restaurant that maintained and slaughtered livestock on the premises, including both diverse avian species, pigs and reptiles	No avian samples were collected at enrolment; two of six close-out samples tested positive for A/H9.	Individual 73–03 did not report any illness episodes, remained seronegative to seasonal antigens and seroconverted to A/H9 between years 1 and 2, with HI titre 5 to 40. Neither of the two other cohort members recruited from the restaurant reported illness episodes or seroconverted.

During the 24 months of monitoring for the BaVi cohort in Hanoi province, a total of 145 episodes of human clinical illness were reported (*n*=101 respiratory swabs from 49 households). Molecular diagnostics revealed one case each of A/H1pdm and A/H3, and three cases of Influenza B. We did not detect evidence for any symptomatic zoonotic transmission of animal IAV. Virological screening of OP swabs from poultry sampled at the study sites (chickens, ducks, quails, ostriches and other poultry) confirmed local transmission of IAV; however, only diseased birds or sudden death cases tested IAV-positive (23 of 40 positive pools from 3 farm sites, representing 69 birds), whereas none of the apparently healthy birds screened positive for IAV (*n*=291 pools from 1020 birds). All IAV M-gene positive detections from diseased birds were confirmed to be A/H5-positive by subtype-specific RT-PCR, and partial HA sequencing identified homology to A/H5 clade 2.3.2.1 c [10]. During 2015, when poultry sudden death cases from Hanoi tested A/H5-positive, we contacted the participating farmers to inform them and offer free access to inactivated A/H5 poultry vaccines. All of the farmers declined to vaccinate their flocks.

Regarding poultry seroprevalence rates, we found higher seropositivity for A/H5 compared to A/H9 at all of the study sites and in both chickens and ducks (detection rates were approximately three times higher for A/H5 compared to A/H9) (Table 3). The majority of A/H9 seropositives were detected among chickens and other avians (mainly geese, pigeons and muscovy ducks) rather than pekin ducks. Variation was observed in IAV seroprevalence across the three provinces, with a marginally higher number of poultry testing positive for A/H9 in northern and central provinces (11.1 and 12.0 % in Hanoi and DakLak, respectively) compared to the southern province (4.1 %, DongThap). These observations are consistent with previous reports of high detection rates for A/H9 at live bird markets along the northern Vietnam/China border [8]. Surveillance data on poultry A/H9 prevalence from live bird markets in central and southern Vietnam are not yet available, as national surveillance programmes have yet to commence routine screening for this low-pathogenic subtype.

**Table 3.** Avian seroprevalence to A/H9 and A/H5 antigens, stratified by province and type of avian species

**Province**	**Subgroup**	**Total A/H9 seropositive (%)**	**Total A/H5 seropositive (%)**	**Seropositive for A/H9 only (%)**	**Seropositive for A/H5 only (%)**	**Seropositive for both A/H9 and A/H5 (%)**	**Log_2_ HIH9 GMT (95 % CI)**	**Log_2_ HIH5 GMT (95 % CI)**
Hanoi	Chicken (*n*=851)	103/851 (12.1)	283/851 (33.6)	56/851 (6.6)	239/851 (28.0)	47/851 (5.5)	3.43 (3.04–3.81)	4.06 (3.63–4.50)
	Duck (*n*=140)	8/140 (5.7)	29/140 (20.7)	2/140 (1.4)	23/140 (16.4)	6/140 (4.3)	3.18 (2.69–3.66)	3.75 (3.29–4.15)
	Other avian (*n*=114)	12/114 (10.5)	22/114 (19.3)	11/114 (9.6)	21/114 (18.4)	1/114 (0.8)	3.39 (2.42–3.85)	3.57 (3.11–4.02)
	**Total: (*n*=1105)**	123/1105 (11.1)	337/1105 (30.5)	69/1105 (6.2)	283/1105 (25.6)	54/1105 (4.9)	3.38 (2.97–3.77)	3.97 (3.55–4.40)
DakLak	Chicken (*n*=478)	84/478 (17.5)	178/477 (37.3)	42/478 (8.8)	136/478 (28.4)	42/478 (8.8)	3.17 (2.63–3.72)	4.13 (3.66–4.59)
	Duck (*n*=79)	1/79 (2.5)	27/79 (34.1)	0/79 (0)	27/79 (34.2)	1/79 (1.2)	2.73 (2.18–3.29)	3.81 (3.29–4.34)
	Other avian (*n*=168)	1/168 (0.6)	28/168 (16.6)	0/168 (0)	27/168 (16.1)	1/168 (0.5)	2.42 (1.71–3.13)	3.31 (2.87–3.72)
	**Total (*n*=725)**	86/725 (12.0)	233/724 (32.1)	42/725 (5.8)	190/725 (26.2)	44/725 (6.0)	2.95 (2.37–3.53)	3.9 (3.47–4.33)
DongThap	Chicken (*n*=820)	49/820 (5.9)	197/820 (24.0)	38/820 (4.6)	44/820 (5.4)	11/820 (1.3)	2.81 (2.26–3.36)	3.58 (3.18–3.99)
	Duck (*n*=250)	5/250 (2.0)	32/250 (12.8)	4/250 (1.6)	31/250 (12.4)	1/250 (0.4)	2.60 (1.98–3.21)	3.15 (2.69–3.61)
	Other avian (*n*=370)	5/370 (1.3)	34/370 (9.2)	3/370 (0.8)	32/370 (8.6)	2/370 (0.5)	2.51 (1.85–3.17)	2.87 (2.33–3.41)
	**Total (*n=*1441)**	59/1441 (4.1)	263/1440 (18.2)	45/1441 (3.1)	249/1441 (17.3)	14/1441 (1.0)	2.69 (2.11–3.29)	3.32 (2.89–3.75)

For farms in Hanoi province at which we confirmed that farm flocks were not vaccinated against A/H5, we noted with surprise a high A/H5 seroprevalence in chickens and other avian species (Table 3). Although ducks are known to frequently sustain completely asymptomatic infections with wild-type HPAI H5, chickens are thought to be exceedingly susceptible to infection, and will typically succumb within 48 h of exposure (although the time to death depends on the route of exposure and viral load). Our finding of widespread A/H5 seropositivity among unvaccinated chickens indicates that the phenomenon of ‘silent’ A/H5 infection may extend beyond ducks. The profile of A/H9 and A/H5 HI reactivity showed a moderate degree of cross-reactive immunity (Table 3), leaving open the possibility that prior exposure to A/H9 may partially mask the disease severity for A/H5. The possibility also remains that the local chicken breeds used in backyard production systems are more resistant to A/H5 infection than the white leghorn breeds typically used for A/H5 virulence assessments.

Our study had several limitations and challenges. These included the small sample size of the human cohort, the lack of differential testing against a comprehensive panel of IAV antigens, and the lack of neutralization tests to assess the functional significance of detected HI antibody titres. For the southern Vietnam study sites, our lack of more detailed information regarding poultry IAV vaccination history also constrained our ability to interpret the IAV seroprevalence results significantly.

In summary, our findings are consistent with reports from the region indicating moderate levels of A/H9 immunity among poultry-exposed populations (3 % seroprevalence), and provide evidence of sporadic asymptomatic avian-to-human A/H9 infections. Our analysis is consistent with expectations that poultry-exposed populations are frequently infected with multiple IAVs, and that mild asymptomatic A/H9 infections likely constitute a measurable fraction of overall IAV transmission. Further studies are required to assess the epidemiological significance of these sporadic A/H9 infections, and to evaluate whether prior A/H9 immunity may influence subsequent response to infection. Sample sets collected from longitudinal cohort studies such as these provide an ideal opportunity for more in-depth analyses of IAV immunity. Determining whether human anti-A/H9 HI antibodies are sufficiently prevalent and persist at high enough titres to influence transmission ecology will require both new prospective cohort studies and more population-based seroepidemiology. New laboratory-based testing methods are now available that allow multiplexed serological screening across large arrays of antigens [20], and these new tools may facilitate the processing of larger serum sets representing diverse types of exposure very significantly. The co-circulation of multiple high- and low-pathogenic avian viruses within Vietnamese farming systems would seem to represent a major risk for zoonotic emergence. However, the fact that seroprevalence levels in Vietnam are within a similar range to those for other non-AIV endemic countries [18], while both A/H5 and A/H9 remain highly endemic in poultry but human cases of avian influenza have not been detected, underscores the complexity of quantifying risk and maintaining vigilant preparedness for problems that have yet to arise.
